# Modified Logistic Regression Approaches to Eliminating the Impact of Response Styles on DIF Detection in Likert-Type Scales

**DOI:** 10.3389/fpsyg.2017.01143

**Published:** 2017-07-07

**Authors:** Hui-Fang Chen, Kuan-Yu Jin, Wen-Chung Wang

**Affiliations:** ^1^Applied Social Sciences, City University of Hong Kong Kowloon Tong, Hong Kong; ^2^Assessment Research Centre, The Education University of Hong Kong Tai Po, Hong Kong

**Keywords:** extreme response styles, logistic regression, likert scale, differential item functioning, mild response style

## Abstract

Extreme response styles (ERS) is prevalent in Likert- or rating-type data but previous research has not well-addressed their impact on differential item functioning (DIF) assessments. This study aimed to fill in the knowledge gap and examined their influence on the performances of logistic regression (LR) approaches in DIF detections, including the ordinal logistic regression (OLR) and the logistic discriminant functional analysis (LDFA). Results indicated that both the standard OLR and LDFA yielded severely inflated false positive rates as the magnitude of the differences in ERS increased between two groups. This study proposed a class of modified LR approaches to eliminating the ERS effect on DIF assessment. These proposed modifications showed satisfactory control of false positive rates when no DIF items existed and yielded a better control of false positive rates and more accurate true positive rates under DIF conditions than the conventional LR approaches did. In conclusion, the proposed modifications are recommended in survey research when there are multiple group or cultural groups.

## Introduction

A considerable number of survey-based studies have reported that the process of mapping answers to response options on Likert-type items may vary between individuals; this is termed response styles (RS; Paulhus, 1991; De Jong et al., 2008; Kieruj and Moors, 2013). RS “may be induced by context effects such as the item format or personality” (Paulhus, 1991), and might further lead to biased conclusions of measurement invariance (Morren et al., 2012). In other words, participants may exhibit a tendency to endorse specific response categories and systematically tick a certain rating option, regardless of item content (Weijters, 2006). As a result, the scale scores might be inflated or reduced and fail to capture participants' true attitudes or beliefs.

Several types of RS have been identified in the literature, and extreme response style (ERS) vs. mild response style (MRS) are the most widely investigated (Harzing et al., 2009). ERS refers to a tendency to use two extremely end points, such as rating categories 0 and 4, when participants rate their agreement to a statement on a five-point scale from 0 (strongly disagree) to 4 (strongly agree). MRS respondents tend to avoid the two “opposite, extreme” categories and consistently choose the middle range of response categories across all items (e.g., 1, 2, and 3). ERS and MRS are mutually exclusive, because they cannot be simultaneously observed on one item. Participants who have a strong tendency to ERS would demonstrate a weak tendency to MRS and vice versa (Jin and Wang, 2014). Therefore, the term of ERS will only be used to refer to the bipolar tendency thereafter.

Degrees of ERS may vary across gender, cultures, educational, or intellectual levels, and age. Although Moors (2012) found that women, compared to men, show a greater tendency to choose two extreme sides of rating options when rating passive/laissez-faire leadership, but Baumgartner and Steenkamp (2001) found no gender differences in marketing surveys. In other studies, Americans were more likely to be ERS respondents than their Chinese peers given the same level of self-esteem (Song et al., 2011), resulting in lower scores found among Chinese and Japanese than Americans and Canadians (Cai et al., 2007; Brown et al., 2009). Educational level is negatively related to ERS (De Beuckelaer et al., 2010): Respondents with a lower education level tended to mainly select middle points of the scale, most likely in order to simplify the task (Weijters, 2006). Meisenberg and Williams (2008) further investigated certain education-related factors, such as high intelligence or self-confidence, and found a suppressing effect on ERS. Regarding the impact of age on ERS, while some studies reported that younger respondents tended to use ERS more often compared to their older counterparts (Rossi et al., 2001; Austin et al., 2006), others claimed that age was non-significant in relation to ERS (Johnson et al., 2005). It is ubiquitous that observed scores on self-reported scales might be contaminated by ERS, and it is necessary to investigate the impact of ERS before conclusions are drawn from measurement scores.

ERS causes problems in interpreting item scores because it creates uncertainty over whether the given answers accurately reflect participants' true opinions. Baumgartner and Steenkamp (2001) argued that ERS constitutes error variance, which might attenuate correlations among variables, and many statistical methods based on correlations are influenced, such as Cronbach's alpha, regression analysis, factor analysis, and structural equation modeling. As a result, the mean levels of responses and the correlations among constructs of interests are biased (Baumgartner and Steenkamp, 2001). Cheung and Rensvold (2000) further pointed out that higher/lower ERS can lead to an increase/decrease in factor loadings. Composite scores of problematic items and good items, which are used to rank participants for personnel selection in organizations or for admission to school, or to compare group differences (e.g., gender and ethnicity differences) for evaluating the effectiveness of curriculum, programs, or educational reforms, lead to misinformed conclusions.

ERS also causes problems in psychometric properties of scales, such as metric and scalar invariance, due to inflated or reduced scale variances (Baumgartner and Steenkamp, 2001). Empirical and simulation studies on ERS have reported that it led to an additional dimension other than the intended-to-be-measured latent trait (Bolt and Johnson, 2009; Morren et al., 2012; Wetzel et al., 2013). All items therefore might be identified as non-invariant when individual groups show different degrees of ERS. In other words, ERS causes measurement non-invariance even when the intercepts, slopes, and variances of the intended-to-be-measured latent trait are invariant across groups (Morren et al., 2012). Standard approaches to testing measurement invariance (e.g., multiple-group confirmatory factor analysis) therefore always conclude a violation of measurement invariance. These findings suggest that standard methods of matching participants based on their latent traits are not appropriate, because the impact of ERS has not been taken into account. While measurement invariance is required to make meaningful comparisons across groups, the influence of ERS should be considered and corrected in data analyses (Weijters et al., 2008).

Although ERS has been recognized in survey research, it is not well-controlled in studies (Van Vaerenbergh and Thomas, 2013) such as the Programme for International Student Assessment (PISA) (Buckley, 2009; Bolt and Newton, 2011). Therefore, the current study aimed at addressing the need to control ERS in survey research and focused on the issue of measurement invariance in Likert- or rating-type scales. We first addressed the reasons why ERS causes misleading conclusions regarding the psychometric properties of Likert-type scales, and then proposed two modified logistic regression methods to eliminate the impact of ERS on the test of measurement invariance. The following sections are organized as follows: introduction to differential item functioning (DIF) assessment, the limitation of standard logistic regression methods for DIF assessment when ERS occurs, the proposed modified logistic regression methods for DIF assessment, the results from a series of simulation studies to compare the performance of the standard and modified methods in DIF assessment, and conclusions, and suggestions for future studies.

## Differential item functioning (DIF) assessment

One prerequisite of comparisons across groups or countries is measurement invariance. DIF assessment is one approach that has been routinely conducted in large-scale assessment programs to ensure that observed or scaled scores are comparable across groups or countries. To meet the assumption of measurement invariance, responses to items should be free from bias inferred by group membership (e.g., gender, ethnicity, or country), so that different performances on tests or questionnaires can be attributed to group difference in the intended-to-be-measured latent trait (e.g., ability, attitude, or interest). However, while researchers often examine the issue of DIF in cognitive assessments, they seldom address it in self-reported inventories, although both types of assessments have been used in large-scale assessment programs to monitor achievement trends and students' learning and living environments on a global basis.

An item is identified as having DIF when participants belonging to different groups have varying probabilities of endorsing that item, given that they have the same levels on the intended-to-be-measured latent trait. In DIF assessment, participants are placed on the same metric using a matching variable, before the performance on the studied item of a focus group and a reference group is compared for DIF. If participants are matched by a biased metric, the subsequent DIF assessment will be misleading (Clauser et al., 1993; Wang, 2004). Usually, the total score of an instrument serves as a matching variable, but this does not always function adequately. Prior studies have shown that only if the total score is a sufficient statistic of or has a strong relationship with the latent trait, and the latent trait distributions are identical or very similar across groups, can the total score serve as a matching variable to yield satisfactory DIF assessment (Bolt and Gierl, 2006; Magis and De Boeck, 2014). Likewise, when participants exhibit ERS, the total score is contaminated and becomes a biased matching variable.

## Logistic regression for DIF detection

The logistic regression (LR) procedure (Swaminathan and Rogers, 1990; Rogers and Swaminathan, 1993) is one of the most popular approaches in DIF assessment. The LR does not require specific forms of item response functions or large sample sizes (Narayanan and Swaminathan, 1994). It is computationally simple and can be easily implemented in commercial software (e.g., SPSS, SAS, and STATA) or free software (e.g., R) without additional effort in terms of computer programming. Therefore, the LR has frequently been used in empirical studies.

The LR was originally developed for dichotomous data. Two extensions of the LR, namely ordinal logistic regression (OLR; French and Maller, 1996; Zumbo, 1999) and logistic discriminant function analysis (LDFA; Miller and Spray, 1993), have been proposed for ordinal responses (e.g., Likert-type scales). When a set of items has *M* ordinal response categories, the OLR for DIF assessment can be expressed as:

(1) $$\begin{array}{l} \text{log }\left[\frac{P\left(Y\;\leq j\right)}{P\;\left(Y\;>j\right)}\right]=\gamma_{0}+\gamma_{1}X+\;\gamma_{2}G+\gamma_{3}XG, \end{array}$$

which is the logarithm of the ratio of the probability of obtaining a score *Y* (*Y* = 0, 1,…, *M*–1) less than or equal to *j* to the probability of receiving a score higher than *j. X* is the total score; *G* is a grouping variable, testing the effect of group for uniform DIF, and *XG* is an interaction between the grouping variable and the total score. If γ_2_ is statistically significantly different from zero (e.g., at the 0.05 nominal level) but γ_3_ is not, then there is a uniform DIF between groups. If γ_3_ is statistically significantly different from zero, a non-uniform DIF is found between groups.

In the LDFA, the observed response *Y* of the studied item, the total score (*X*), and their interaction (*XY*) are used to predict the probability of being in a specific group (*G*). The full model of the logistic function is defined as:

(2) $$\begin{array}{l} \text{log }\left[\frac{P\;\left(G=1\right)}{P\;\left(G=0\right)}\right]=\alpha_{0}+\alpha_{1}X+\;\alpha_{2}Y+\alpha_{3}XY. \end{array}$$

Likewise, if α_2_ is significantly different from zero but α_3_ is not, then there is a uniform DIF between groups. A non-uniform DIF is found if α_3_ is significantly different from zero.

Although the OLR and LDFA were developed to detect DIF for ordinal responses within the framework of LR, they are exactly independent approaches. It is noticeable that the grouping member serves as a predictor in the OLR, as in the conventional LR approach for dichotomous data, but becomes an outcome variable in the LDFA. Operationally, the LDFA may be preferred by practitioners because it keeps the feature of simplicity in the binary LR where the outcome is binary (0 and 1).

Compared to studies on dichotomous items, fewer studies have investigated the performance of the above approaches in detecting DIF items in polytomous data. Most have suggested that all approaches show similar power in detecting uniform DIF (Kristjansson et al., 2005) and that their performance is influenced by sample sizes, DIF magnitude, and latent trait distributions (Zwick et al., 1997; Ankenmann et al., 1999; Wang and Su, 2004; Kristjansson et al., 2005; Su and Wang, 2005). High item slope parameters and large group ability differences usually cause inflated Type I error rates (Zwick et al., 1997; Ankenmann et al., 1999). Most procedures yield increased power with increased item slope (Kristjansson et al., 2005).

The reason why ignoring ERS would lead to erroneous judgment in DIF assessment could be explained by using the ERS model with generalized partial credit modeling (ERS-GPCM) (Jin and Wang, 2014). The ERS-GPCM is expressed as:

(3) $$\begin{array}{l} \text{log }\left[\frac{P_{nij}}{P_{ni\left(j-1\right)}}\right]=\beta_{i}\left[\theta_{n}-\left(\delta_{i}+\omega_{n}\tau_{ij}\right)\right], \end{array}$$

where *P*_*nij*_ and *P*_*ni*(*j*−1)_ are the probabilities of selecting options *j* and *j–*1 for person *n*, respectively; θ_*n*_ is the latent trait of person *n* and is assumed to follow a normal distribution with a mean of zero and variance of $\sigma_{\theta }^{2}$; ω_*n*_ denotes the ERS tendency of person *n* and is assumed to follow a log-normal distribution with a mean of zero and variance of $\sigma_{\omega }^{2}$; δ_*i*_; and β_*i*_ are the mean location parameter and the slope parameter of item *i*, respectively; and τ_*ij*_ is the *j*th threshold of item *i*. In addition, ω is assumed to be independent of θ, and both are random variables. As pointed out by Jin and Wang (2014), ω_*n*_ can be treated as a personal factor for controlling the occurrence of ERS. A participant with a larger ω is more likely to choose middle response categories, whereas a participant with a smaller ω is more likely to choose extreme response categories. A larger $\sigma_{\omega }^{2}$ implies that participants in a group showed more heterogeneous ERS. When $\sigma_{\omega }^{2}$ is 0, ω will be 1 for all participants (i.e., the ERS levels are identical for all participants); therefore, Equation (3) reduces to the generalized partial credit model (Muraki, 1992). In particular, the assumption that the ERS tendency and the latent trait are compensatory, which is questionable but required in other models for ERS (e.g., Bolt and Johnson, 2009; Johnson and Bolt, 2010; Bolt and Newton, 2011), is not made in the ERS-GPCM.

**Figure 1:**
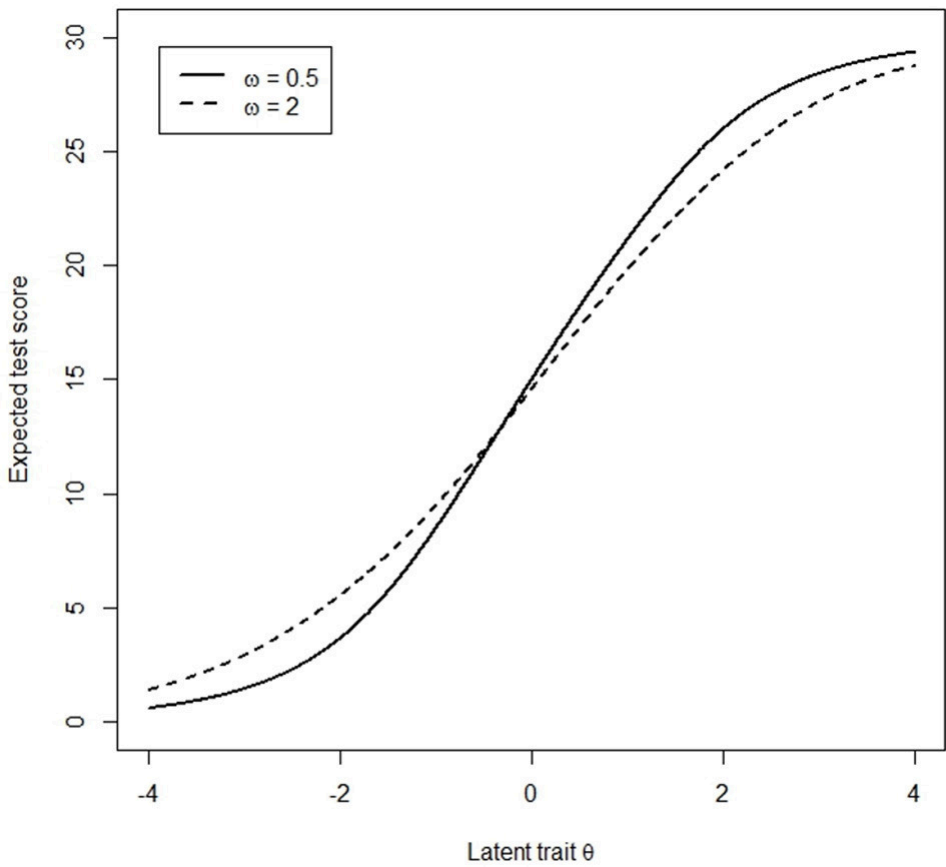
Cumulative test characteristic curves for respondents with different ERS levels.

Assume that participants are from two groups, with a stronger tendency to endorse extreme responses (ω = 0.5) and a tendency to choose the middle range of categories (ω = 2) respectively, termed the focal group and the reference group, respectively. Given the same level of latent trait, participants with different magnitudes of ω have different expected total scores (Figure 1). Thus, the correspondence between the latent trait and the total score is poor, such that the total score is no longer a good indicator of the latent trait when ERS exists. For instance, participants with ω of 0.5 and θ of −2 will be matched with participants with ω of 2 and θ of −1.5 because their total scores are the same in standard DIF assessment. As a result, the total score is not a valid matching variable in standard DIF assessment.

Because ERS is common and threatens test validity, leading to biased comparisons among individuals, it should be taken into consideration properly. To the best of our knowledge, none of the existing studies have considered the effect of ERS with the logistic regression approaches in DIF assessments. One noticeable difference between the current study and previous work (e.g., Bolt and Johnson, 2009; Morren et al., 2012) is the specification of whether a measurement model is used. In the studies of Morren et al. (2012) and Bolt and Johnson (2009), a measurement model, which incorporated not only the mechanism of DIF but also ERS, was implemented to fit to data. Conversely, a strategy of jointly using the summation and variance of item scores as matching variables in the OLR or LDFA method is proposed in our study. In other words, no measurement model is specified.

This study focused on the influence of group difference in ERS on DIF detection, because participants from different cultures or groups may have different degrees of tendencies to choose extreme response categories (e.g., Chen et al., 1995; Hamamura et al., 2008; Song et al., 2011; Kieruj and Moors, 2013). It was expected that group difference in ERS would cause inflated false positive (FP) rates of DIF detection in standard LR, and that the modified LR would yield well-controlled FP rates. Three simulation studies were conducted in this paper. Simulation study 1 examined the influence of difference in ERS between the focal and reference groups on the performance of standard LR in detecting (uniform) DIF; simulation study 2 demonstrated the superiority of the modified LR; and simulation study 3 evaluated the performances of the standard and modified LR when tests had multiple DIF items. Finally, gender DIF was investigated on an anxiety scale and results from the standard and modified LR methods were compared.

## Simulation study 1

### Design

In the first simulation study, we examined the performance of the standard OLR and the LDFA in detecting uniform DIF when two groups of participants exhibited different levels of ERS on average. Consequently, γ_3_ in Equation (1) and α_3_ in Equation (2) were constrained at 0. Data were generated from Equation (3). A sample of 500 participants in each group, answering a four-point scale, was generated. There were either 10 or 20 items. The settings for item parameters were as follows: (a) mean location parameters were generated from uniform (−2, 2); (b) slope parameters were generated from log-normal (0, 0.3^2^); (c) the three threshold parameters were set at −0.6, 0, and 0.6 for all items. For the focal and reference groups, the latent trait θ was randomly generated from the standard normal distribution. The ERS level (i.e., ω) was generated from the log-normal distribution with identical variance of 0.6 but with different means. Using the log-normal scale, the mean of ω was set at 0, 0.2, and 0.3 for the reference group and 0, −0.2, and −0.3 for the focal group, respectively. Thus, mean differences of 0, 0.4, and 0.6 were used to indicate no, moderate, and large differences in ERS between groups (hereafter to be referred to as no, moderate, and large difference in ERS, respectively). Each condition consisted of 1,000 replications. All items in a test were assessed for DIF using the OLR and LDFA. Here, the item location and slope parameters were set to be identical across the groups (i.e., all items were DIF-free), so the FP rate of an item was computed as the percentage of times the item was mistakenly identified as having DIF across the 1,000 replications.

### Results

The left panels in Table 1 show the performance of the standard OLR and the LDFA when tests had 10 items. Both methods performed well when there was no group difference in ERS, so that the FP rate for all items was around the nominal level of 0.05. However, when differences were moderate or large, both methods yielded severely inflated FP rates. When the difference was moderate, the mean FP rate was 0.19 and 0.11 for the standard OLR and the LDFA, respectively; when it was large, mean FP rate was 0.31 and 0.18 for the two methods, respectively. Similar patterns were found when tests had 20 items, but the inflation on the FP rates became more serious (see the left panels in Table 2), because the matching variable (total score) was more severely contaminated by ERS. In general, when the group difference in ERS was large, the chance of most items being misidentified as having DIF increased by more than 20% on average.

**Table 1:** False positive rates (‰) in a 10-item test.

	**Generated values**		**OLR**			**LDFA**			**OLR-m**			**LDFA-m**		
**Item**	**β**	**δ**	**I**	**II**	**III**	**I**	**II**	**III**	**I**	**II**	**III**	**I**	**II**	**III**
1	0.634	−1.112	41	46	62	43	52	58	45	50	58	43	48	46
2	1.372	1.483	52	614	903	51	337	617	55	111	178	55	71	85
3	0.962	−1.173	57	223	430	51	106	199	53	54	63	53	56	68
4	0.655	1.674	63	111	167	48	65	86	58	83	98	59	55	62
5	1.198	−0.046	37	88	143	40	51	70	41	42	44	39	41	46
6	0.872	0.447	62	495	808	50	238	439	44	131	219	46	53	64
7	0.959	1.064	63	157	316	52	85	122	65	65	72	51	60	57
8	0.745	0.074	48	50	56	54	48	53	48	55	65	47	49	53
9	1.130	−0.813	43	44	51	40	47	52	48	44	50	41	42	45
10	1.076	−1.249	45	113	179	43	89	139	53	56	57	54	58	58
Average			51.1	194.1	311.5	47.2	111.8	183.5	51.0	69.0	90.0	48.8	53.3	58.4

*I, II, and III denote that the mean differences in the magnitude of ERS were set at 0, 0.4, and 0.6, respectively; significance level at 0.05*.

**Table 2:** False positive rates (‰) in a 20-item test.

	**Generated values**		**OLR**			**LDFA**			**OLR-m**			**LDFA-m**		
**Item**	**β**	**δ**	**I**	**II**	**III**	**I**	**II**	**III**	**I**	**II**	**III**	**I**	**II**	**III**
1	1.127	−1.112	58	366	643	58	193	350	48	87	116	55	63	68
2	0.949	1.483	64	492	800	62	284	509	58	103	131	52	56	57
3	0.909	−1.173	54	256	469	46	137	244	50	50	61	49	51	57
4	0.719	1.674	48	364	620	42	215	373	37	58	71	39	44	48
5	0.792	−0.046	51	54	54	57	55	56	53	58	61	59	53	56
6	0.589	0.447	56	87	124	58	68	89	61	51	54	58	62	63
7	0.560	1.064	52	160	268	47	103	161	52	55	52	49	54	62
8	1.581	0.074	57	80	108	56	65	74	58	54	50	60	58	48
9	1.226	−0.813	58	310	518	49	161	270	56	76	92	52	61	61
10	0.505	−1.249	45	99	151	46	66	100	49	52	46	48	54	59
11	0.651	−1.677	49	217	412	42	120	213	46	53	60	51	54	67
12	0.716	0.954	58	188	341	56	121	201	53	63	63	58	66	72
13	1.315	−0.235	53	60	69	54	57	65	56	54	56	50	56	55
14	1.340	−1.367	61	651	920	57	342	609	58	112	186	49	45	45
15	0.623	1.520	77	243	445	77	144	250	65	61	62	70	69	73
16	1.063	−0.904	64	251	457	56	130	223	57	66	84	57	58	70
17	0.568	−0.343	51	51	55	46	54	57	54	48	51	45	48	51
18	1.247	−0.816	65	290	515	58	150	276	62	72	93	50	60	63
19	1.398	0.515	54	300	524	53	167	277	51	87	88	52	48	53
20	1.250	0.319	53	151	246	49	97	133	59	59	53	52	52	54
Average			56.4	233.5	387.0	53.5	136.5	226.5	54.2	66.0	76.5	53.4	55.6	60.3

*I, II, and III denote that the mean differences in the magnitude of ERS were set at 0, 0.4, and 0.6, respectively; significance level at 0.05*.

**Figure 2:**
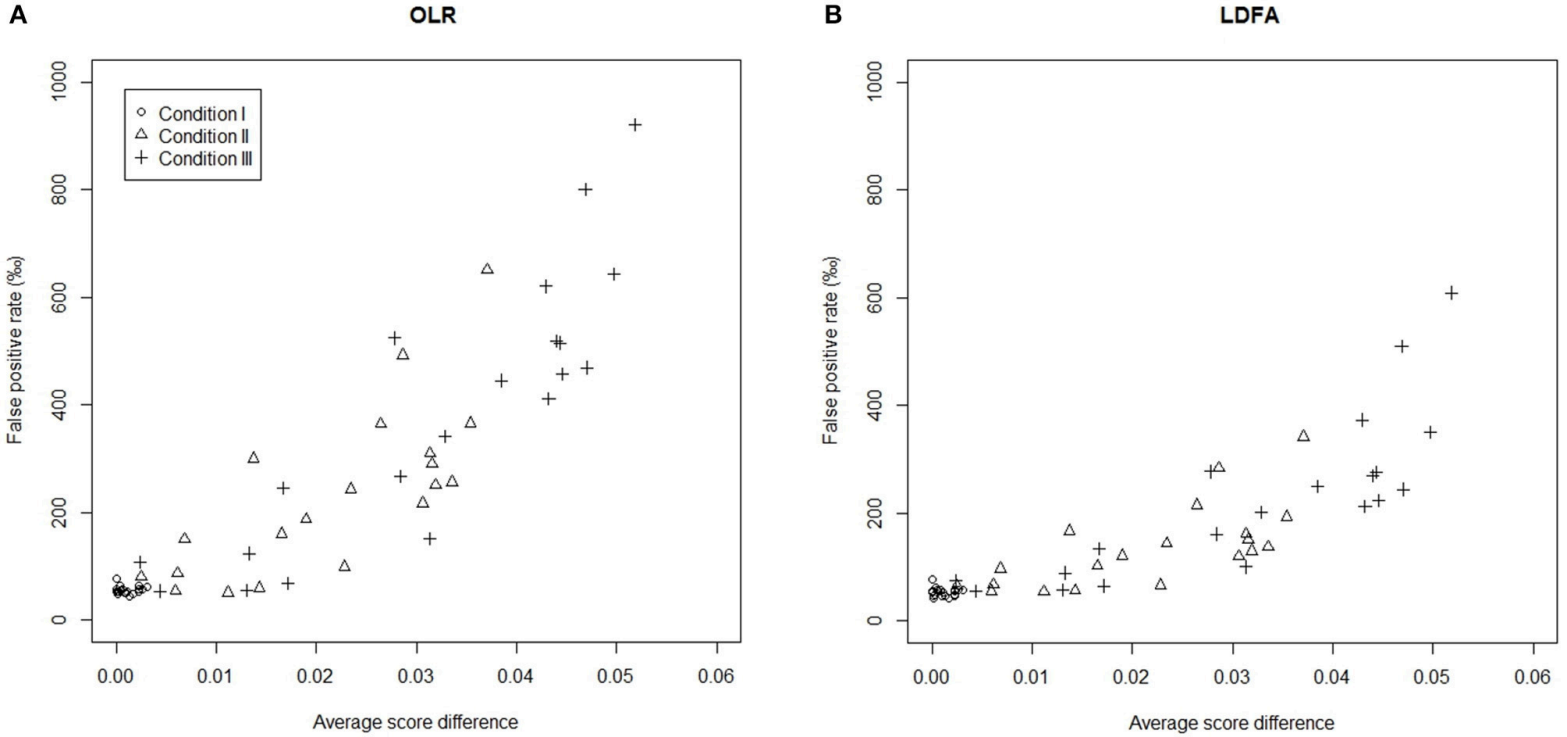
Relationships between the average score difference (in absolute value) and the false positive rate in a 20-item test. **(A)** OLR and **(B)** LDFA. Conditions I, II, and III denote that the mean differences in the magnitude ofERS were set at 0, 0.4, and 0.6, respectively.

To better understand why a group difference in ERS might influence DIF assessment, a Monte Carlo procedure was conducted to compute the average score difference (ASD) between the two groups of each item. A total of 100,000 simulees were generated, given distributions of θ and ω for the focal (and reference) group, and the expected scores on items were computed. The ASD was obtained by calculating the difference between the mean scores for the focal and reference groups. Figure 2 includes two scatter plots showing a positive relationship between the ASD (in absolute value) and FP rate for the standard OLR and LDFA. In the 20-item test, for example, the ASD on item 1 was 0.00, 0.04, and 0.05, respectively when there was no, moderate, and large group difference in ERS. When there was no difference between groups, the ASD on all items approximated to zero, suggesting that the usage of total score as the only matching variable was still effective. On the other hand, under the conditions with moderate and large difference in ERS, non-zero ASD was found, which therefore decreased the appropriateness of total score. In addition, the larger the ASD of an item, the higher its probability of being flagged as DIF. Apparently, both standard LR methods failed to consider the ASD caused by the group difference in ERS, leading the FP rates to become out of control.

It is concluded that the total score contaminated by ERS is not an appropriate matching variable for both LR methods. The standard OLR performs worse than the standard LDFA when there is an ERS effect. The group difference in ERS causes measurement non-invariance and invalidates the standard OLR and LDFA procedures in that DIF-free items are often misidentified as DIF items. Modifications of the standard OLR and LDFA are needed to partial out the group difference in ERS and improve the DIF assessment.

## Simulation study 2

### Design

A new class of LR procedures for eliminating the impact of a difference in ERS on DIF assessment was proposed, and their performance was evaluated in simulation study 2. As indicated by Jin and Wang (2014), the tendency of ERS is related to the extent of dispersion of item scores. Scores of a participant with ERS would be more extreme and their variance would be larger than those of a participant with MRS. Therefore, in order to rule out the interference of ERS/MRS in DIF assessment, the dispersion of item scores should be taken into consideration when the matching variable is constructed; here, the variance of item scores seemed to be a good representative of the ERS effect. The modified OLR (OLR-m) can be expressed as follows:

(4) $$\begin{array}{l} \text{log }\left[\frac{P\left(Y\;\leq j\right)}{P\;\left(Y\;>j\right)}\right]=\gamma_{0}+\gamma_{1}X+\gamma_{2}G+\gamma_{3}XG+\gamma_{4}S+\gamma_{5}XS, \end{array}$$

where *S* indicates the variance of individuals' item scores, *XS* is the interaction between the total score and the variance, and others are the same as stated previously. The modified LDFA (denoted as LDFA-m) then becomes:

(5) $$\begin{array}{l} \text{log }\left[\frac{\text{P }\left(\text{G}=1\right)}{\text{P }\left(\text{G}=0\right)}\right]=\;\alpha_{0}+\alpha_{1}X+\alpha_{2}Y+\alpha_{3}XY+\alpha_{4}S+\alpha_{5}XS. \end{array}$$

The inclusion of the predictors of *S* and *XS* is to partial out the ERS effect such that γ_2_ or γ_3_ in Equation (4), and α_2_ and α_3_ in Equation (5), can be used to properly detect uniform or non-uniform DIF. Because we focus on uniform DIF, γ_2_ and γ_3_ in Equation (4) and α_2_ and α_3_ in Equation (5) are constrained at zero. To evaluate whether OLR-m and LDFA-m outperformed the standard OLR and LDFA, the same conditions as in simulation study 1 were examined to see if the modified models could yield an acceptable FP rate.

### Results

The right panels in Tables 1, 2 show that, as the difference in ERS increased, the FP rates for the OLR-m and the LDFA-m rose slightly. However, the FP rate was generally well-controlled around the 0.05 level, except when the OLR-m was adopted in 10-item tests with a large ERS effect, where the FP rate was slightly inflated. The mean FP rate across items in the OLR-m ranged from 0.051 to 0.090 when tests had 10 items, and 0.054–0.076 when tests had 20 items. The LDFA-m yielded a mean FP rate between 0.049 and 0.058 when tests had 10 items, and 0.053 and 0.060 when tests had 20 items. It seems that the LDFA-m outperformed the OLR-m in controlling the FP rate. Although the OLR-m yielded a slightly inflated FP rate when the difference in ERS was large, generally speaking, both modified procedures significantly outperformed their counterparts (standard OLR and LDFA).

## Simulation study 3

In simulation studies 1 and 2, it was assumed that all items in a test were DIF-free. In reality, tests often (if not always) contain DIF items. In simulation study 3, we evaluated the performances of the standard and modified OLR and LDFA when tests had uniform DIF items. We set 20% of items as DIF items and manipulated two DIF patterns, balanced and unbalanced. In the balanced DIF condition, half of the DIF items favored the focal group and the other half favored the reference group; in the unbalanced DIF condition, all DIF items favored the focal group. In practice, most DIF patterns in a test will fall between these two extreme patterns. The differences of mean location parameters for DIF items were set at a constant of 0.2 logit. It was expected that the OLR-m and LDFA-m would yield a good control of FP rate and a high true positive (TP) rate, which was computed as the percentage of times a DIF item was correctly identified as having DIF across the 1,000 replications.

### Results

**Figure 3:**
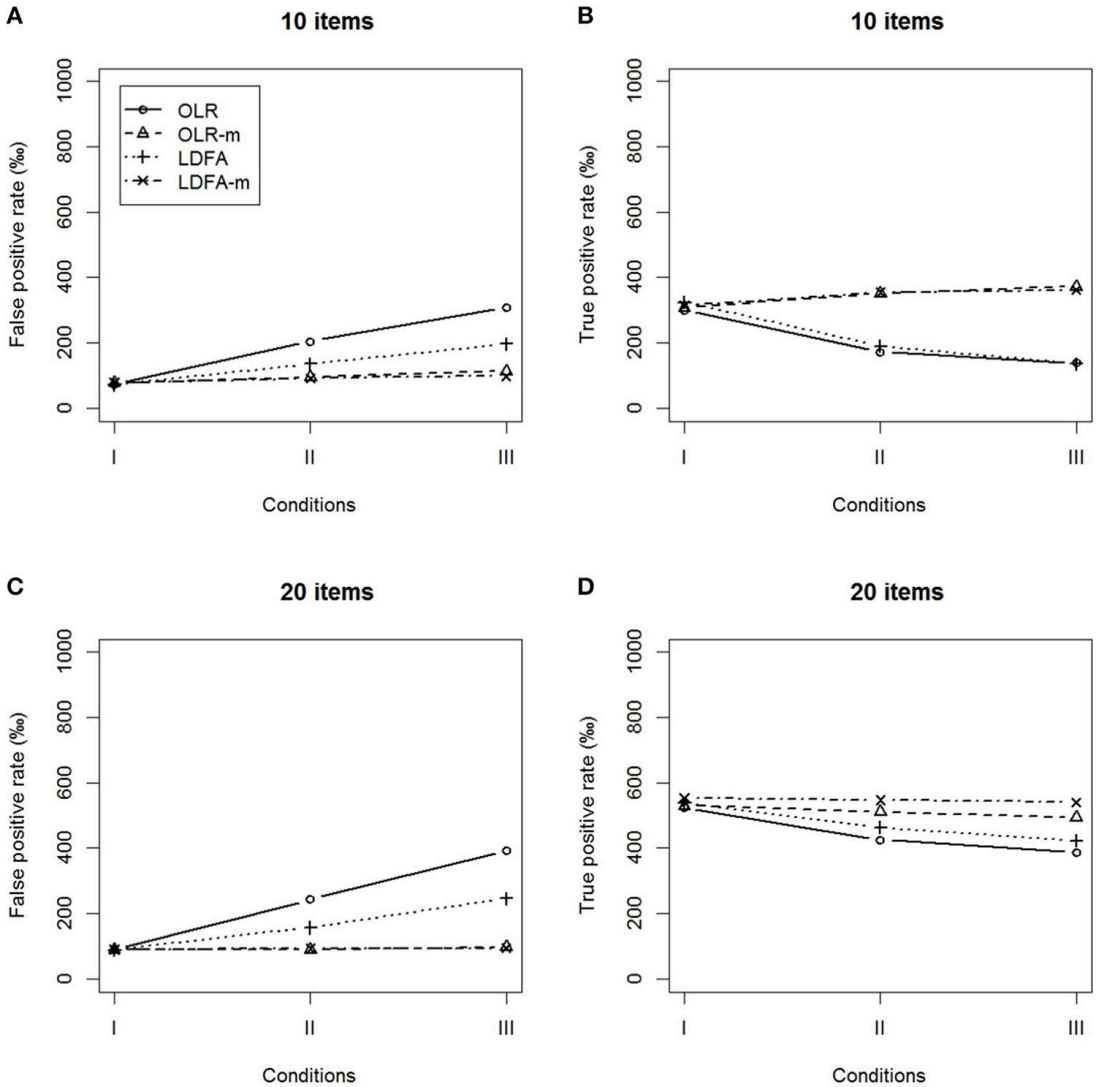
False positive rates and true positive rates under the conditions of unbalanced DIF. **(A,B)** 10 items and **(C,D)** 20 items.

**Figure 4:**
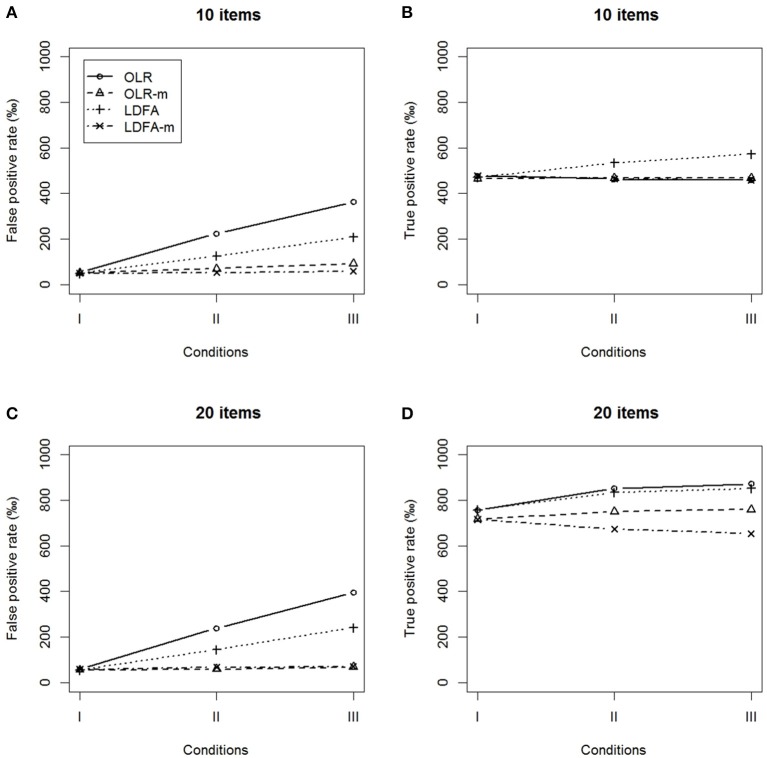
False positive rates and true positive rates under the conditions of balanced DIF. **(A,B)** 10 items and **(C,D)** 20 items.

We compared the FP and TP rates of the OLR-m and LDFA-m with those of the standard OLR and LDFA, as shown in Figures 3, 4. When the DIF pattern was unbalanced, both the OLR-m and LDFA-m showed a better control of FP rate and yielded a higher TP rate across all conditions than the standard OLR and LDFA. The standard OLR and LDFA performed more poorly in terms of inflated FP rates and deflated TP rates when there was a moderate or large difference in ERS. More than 10% of DIF-free items were identified as having DIF in the standard OLR and LDFA, while the TP rate decreased substantially when there was a large difference in ERS.

Similar results were found in the balanced DIF condition. The OLR-m and LDFA-m showed a better control of the FP rates and yielded higher TP rates than their counterparts, regardless of the magnitude of the ERS effect. The standard approaches identified more than 10% of DIF-free items as having DIF and yielded a lower TP rate as the magnitude of difference in ERS increased. In summary, the modified approaches outperformed their standard counterparts when tests had DIF items.

## An empirical example

Data for this example came from the anxiety scale of the patient-reported outcomes measurement information system (PROMIS) (Pilkonis et al., 2011). The PROMIS was developed by the National Institute of Health and provides item banks for the measurement of patient-reported health outcomes (Cella et al., 2007; Buysse et al., 2010). The anxiety scale focuses on fear, anxious misery, hyperarousal, and somatic symptoms related to arousal (Cella et al., 2010). Participants included 369 males and 397 females, who responded to 29 five-point Likert-type items. The dataset can be accessed in the lordif R package (Choi et al., 2011). In the anxiety data, males and females, on average, endorsed 17.91 and 16.29 extreme responses, respectively, implying a gender difference in ERS (*p* = 0.01). The four DIF detection methods, including the standard/modified OLR and LDFA, were applied to detect whether there were items functioning differentially between males and females.

Table 3 summarizes the results of different DIF detection methods on the anxiety scale. The four methods detected seven to eight out of 29 items as DIF items. In particular, items 6, 19, and 20 were consistently flagged as DIF items among these approaches. When comparing the standard and modified approaches, discrepancies were found. The LDFA seemed to yield larger discrepancies compared to the other approaches. For instance, only the LDFA identified items 15, 28, and 29 as DIF items. The inconsistency between the standard and modified approaches may be a result of whether ERS was considered in the implemented DIF detection method. According to the simulation findings, the result of DIF detection for the two modified LR methods would be more reliable.

**Table 3:** Results of the four DIF detection methods in the anxiety scale.

**Item**	**Description**	**OLR**	**LDFA**	**OLR-m**	**LDFA-m**
1	I felt fearful				
2	I felt frightened	+		+	+
3	It scared me when I felt nervous				
4	I felt anxious				
5	I felt like I needed help for my anxiety				
6	I was concerned about my mental health	−	−	−	−
7	I felt upset	+	+		+
8	I had a racing or pounding heart				
9	I was anxious if my normal routine was disturbed	−		−	
10	I had sudden feelings of panic				
11	I was easily startled				
12	I had trouble paying attention			−	−
13	I avoided public places or activities				
14	I felt fidgety				
15	I felt something awful would happen				
16	I felt worried		+		
17	I felt terrified				
18	I worried about other people's reactions to me				
19	I found it hard to focus on anything other than my anxiety	−	−	−	−
20	My worries overwhelmed me	+	+	+	+
21	I had twitching or trembling muscles	−		−	
22	I felt nervous		+		+
23	I felt indecisive				
24	Many situations made me worry				
25	I had difficulty sleeping				
26	I had trouble relaxing				
27	I felt uneasy				
28	I felt tense		+		
29	I had difficulty calming down		−		

*+ indicates DIF items favoring females; − indicates DIF items favoring males*.

## Discussion

Non-negligible individual differences in the mapping process of response categories are a critical concern in cross-group and cross-cultural studies, as they lead to measurement non-invariance. In the present research, we investigated the impact of differences in ERS on DIF assessment and proposed a new class of procedures to eliminate their effect and further improve the performance of the LR approach. As expected, even when item location and slope parameters were identical between the reference and focal groups (DIF-free), the existence of ERS rendered total scores inappropriate for service as a matching variable. The standard LR approaches therefore yield an inflated FP rate and a deflated TP rate. In addition, compared to the standard LDFA, the standard OLR seems to be more vulnerable to ERS, with evidence of a more inflated FP rate. A total score, being a matching variable in standard LR approaches, is contaminated by ERS so seriously that the performance of this method in DIF assessment is unreliable.

In contrast, the modified LDFA and OLR, with the inclusion of item score variance and its interaction with total score as predictors, can partial out the ERS effect so that the subsequent DIF assessment becomes appropriate. Findings from the simulation studies have suggested that the OLR-m and LDFA-m not only outperform their standard counterparts, but also maintain a good control of FP rate and yield a high TP rate in DIF detection even when ERS exists.

A concern was raised by a reviewer regarding why score variance is a better indicator than score standard deviation (*SD*) as a measure of ERS. We compared the proposed approaches with the ones including standard deviation (*SD*) of scores as a predictor. The results showed that the absolute value of the correlation between variances of scores and ω was −0.42, slightly higher than the one (−0.40) of *SD* of scores and ω. In other words, the ω in the ERS-GPCM has a stronger relationship with score variance than with score *SD*. It might be the main reason why the inclusion of score variances in the modified LR approach performed better than the ones with score *SD*s. Therefore, we suggested the modified approaches with the inclusion of score variances.

ERS is reasonably stable across times and scales. Wetzel et al. (2016) assessed personality, vocational interests, and social interaction anxiety of students in German high schools and reported the stability of ERS over a period of 8 years. Weijters et al. (2008) proposed a longitudinal multiple-indictors, multiple-covariates (MIMIC) model to investigate the impact of demographic information on RS and detect the potential biases yielded by the same. Given the simplicity of our approaches and the reduced requirements in terms of sample sizes, it is suggested that future studies implement the modified methods to partial out the ERS effect in longitudinal or multiple-scale studies. Moreover, although the measurement model was the ERS-GPCM, it can be other measurement model for ERS, such as the multidimensional nominal response model (Thissen-Roe and Thissen, 2013; Falk and Cai, 2016). Future studies can be conducted to investigate how the modified LDFA and OLR method will perform when the data are simulated from other measurement models.

The strategy of considering ERS may be applied to other existing DIF detection methods, such as the generalized Mantel-Haenszel (GMH; Mantel and Haenszel, 1959; Holland and Thayer, 1988) and the Mantel method (Mantel, 1963). The Mantel and the GMH usually perform as adequately as the LDFA and the OLR in uniform DIF detections (Kristjansson et al., 2005), even when 40% of items are DIF items in a scale (Wang and Su, 2004). Furthers studies are required to investigate how the GMH and the Mantel work in DIF assessment when ERS occurs.

The present study focused on the assessment of uniform DIF and did not evaluate the assessment of non-uniform DIF. Future studies can extend our approaches to non-uniform DIF assessment and compare their performance with the GMH and Mantel approaches. Previous studies have reported controversial findings regarding the performance of the LR and MH methods in polytomous data (e.g., Hidalgo and López-Pina, 2004; Kristjansson et al., 2005). It is important to revisit these issues when ERS occurs.

The study can be extended to two conditions: the impact of different latent ability distributions of the reference and the focal groups and the studied item slope parameter. The literature has reported a large difference in the ability distribution, with high item discrimination being relevant to poor performance in DIF assessments (Kristjansson et al., 2005; Su and Wang, 2005). The performance of modified approaches might also be influenced by these two factors and it is worth investigating this further before making recommendations for the application of research.

## Author contributions

HC contributed to the conception, design, and analysis of data as well as drafting and revising the manuscript; KJ contributed to the conception, design, analysis of data, and critically revising the manuscript; WW contributed to the conception, design, and revising the manuscript.

### Conflict of interest statement

The authors declare that the research was conducted in the absence of any commercial or financial relationships that could be construed as a potential conflict of interest.
