# Corrigendum: Modified Logistic Regression Approaches to Eliminating the Impact of Response Styles on Differential Item Functioning Detection in Likert-Type Scale

**DOI:** 10.3389/fpsyg.2018.01122

**Published:** 2018-06-26

**Authors:** Hui-Fang Chen, Kuan-Yu Jin, Wen-Chung Wang

**Affiliations:** ^1^Applied Social Sciences, City University of Hong Kong, Kowloon Tong, Hong Kong; ^2^Assessment Research Centre, The Education University of Hong Kong, Tai Po, Hong Kong

**Keywords:** extreme response styles, logistic regression, likert scale, differential item functioning, mild response style

There is an error in the Funding statement. The correct number for **(the Research Grants Council of the Hong Kong Special Administrative Region, China)** is **(CityU 21615416)**. The authors apologize for this error and state that this does not change the scientific conclusions of the article in any way.

The original article has been updated.

## Conflict of interest statement

The authors declare that the research was conducted in the absence of any commercial or financial relationships that could be construed as a potential conflict of interest.

